# Scent detection of *Brucella abortus* by African giant pouched rats (*Cricetomys ansorgei*)

**DOI:** 10.1186/s12917-023-03786-y

**Published:** 2023-10-30

**Authors:** Raphael Mwampashi, Ellie Cutright, Cynthia D. Fast, Bassirou Bonfoh, Rudovick R. Kazwala, Coletha Mathew

**Affiliations:** 1 College of Veterinary Medicine and Biomedical Sciences, Sokoine University of Agriculture, Morogoro, Tanzania; 2 APOPO, SUA-APOPO Rodent Project, Tiba Road, PO Box 3078, Morogoro, Tanzania; 3 Evolutionary Ecology Group, Department of Biology, University of Antwerp, Universiteitsplein 1, Wilrijk, 2610 Belgium; 4 Rutgers Center for Cognitive Science, 152 Frelinghuysen Road, Piscataway, NJ USA; 5 Swiss Centre for Scientific Research, Abidjan, Côte d’Ivoire

**Keywords:** Brucellosis, *Brucella-abortus*, Scent detection, African giant pouched rat, *Cricetomys*, Scent-based diagnostic

## Abstract

**Background:**

Brucellosis is a contagious zoonosis caused by bacteria of the genus *Brucella.* While the disease has been eradicated in most developed countries, it remains endemic in sub–Saharan Africa where access to reliable diagnostics is limited. African giant pouched rats (*Cricetomys ansorgei*) have been trained to detect the scent of *Mycobacterium tuberculosis* to increase case detection in sub-Saharan Africa. Given the similar diagnostic challenges facing brucellosis and tuberculosis, we explored the feasibility of training African giant pouched rats to detect *Brucella*.

**Results:**

After 3 months of training, rats reliably identified cultured *Brucella*, achieving an average sensitivity of 93.56% (SD = 0.650) and specificity of 97.65% (SD = 0.016). Rats readily generalized to novel, younger *Brucella* cultures that presumably generated a weaker volatile signal and correctly identified at least one out of three fecal samples spiked with *Brucella* culture during a final test of feasibility.

**Discussion:**

To our knowledge, these experiments are the first to demonstrate *Brucella* emits a unique odor profile that scent detection animals can be trained to identify. Importantly, cultured *E. coli* samples were included throughout training and test to ensure the rats learned to specifically identify *Brucella* bacteria rather than any bacteria in comparison to bacteria-free culture medium. *E. coli* controls therefore served a crucial function in determining to what extent *Brucella abortus* emits a unique odor signature. Further research is needed to determine if a *Brucella*-specific volatile signature is present within clinical samples. If confirmed, the present results suggest trained rats could serve as a valuable, novel method for the detection of *Brucella* infection.

## Background

Brucellosis is a contagious zoonosis affecting domestic and wild animals worldwide with over 500,000 new human cases annually [1]. It is caused by a bacterium from the genus *Brucella* with 12 species identified to date [2]. It is transmitted mostly through contact with an infected animal or their products [3]. Brucellosis is characterised mainly by abortion, orchitis, and loss of productivity [4] in animals and febrile illness with flu-like symptoms, intermittent fever, and musculoskeletal pain in humans [5, 6]. Severe and complicated cases of human brucellosis are characterized by neurological symptoms and endocarditis which can be fatal [7]. However, clinical signs are highly variable and non-specific, making it difficult to diagnose based on clinical symptoms, especially in regions where other febrile illnesses, like malaria, coexist. While the disease has been eradicated in most developed countries, it remains endemic in sub–Saharan Africa, India, central Asia, Mexico, and central and southern America [8]. The true burden of the disease in developing countries is underestimated due to poor surveillance systems [7] and lack of accurate diagnostic tools [9]. Early and accurate diagnosis is necessary to guide treatment in humans and establish control measures in animals.

Several diagnostic tests are available for brucellosis, both in humans and animals. They are broadly divided into serological, bacteriological, or molecular-based assays [10]. Serological tests include the Rose Bengal Test (RBT), serum agglutination test (SAT), complement fixation test (CFT), and ELISAs. RBT is used as initial screening assay in both humans and animals as recommended by the World Health Organization (WHO) and the World Organization for Animal Health (WOAH) [4] with confirmation achieved using ELISAs. However, serological methods have varying specificities and sensitivities and are not capable of differentiating between *Brucella* spp responsible for the infection, thereby limiting impact for treatment and control programs. Most of these tests use antigens derived from smooth LPS, excluding antibodies from *Brucella* rough strains. Among the smooth strains detected, it is impossible to differentiate vaccinated animals from infected ones. For these reasons, serological tests are not recommended for stand-alone diagnosis [11–13]. While PCR-based molecular assays can demonstrate presence of *Brucella* DNA in a sample, their sensitivity is influenced by the DNA extraction protocol used [14] and they are expensive and require skilled personnel and sophisticated machines [15]. These aspects limit widespread use in resource-poor countries, which shoulder the greatest disease burden. Bacteriological methods used to detect the organism in pure culture set the “Gold Standard” for *Brucella* diagnosis because they can also allow biotyping [16]. However, this technique poses occupational risk to laboratory personnel, requiring personal protective equipment and access to biosafety cabinets to avoid environmental contamination. Bacteriological tests also require up to 14 days for results, making them less practical [17] for determining patient treatment.

African giant pouched rats (*Cricetomys ansorgei*) are indigenous to sub-Saharan Africa and have a highly developed sense of smell. These rats have been successfully trained to detect the odor signature of explosive compounds used in landmines, enabling them to safely locate buried explosives (with bodyweights under 1.5 kg, the rats do not apply enough pressure to detonate a landmine) in former conflict zones in Angola, Mozambique, and Cambodia [18]. Research that began in 2001 has also established a role for trained *C. ansorgei* in the detection of diseases, notably tuberculosis (TB) [19, 20]. Since 2007, the accuracy and efficiency of the TB-detection rats has been evaluated in field conditions with studies [21–23] revealing the rats are more cost-efficient and sensitive than sputum smear microscopy (a commonly used diagnostic tool that is often the only available tool in resource-limited settings), particularly among patient populations difficult to diagnose with traditional methods (such as people living with HIV and children [22]). Ongoing research positions the rats as a second-line diagnostic tool in Tanzania and Ethiopia where sputum samples that have undergone sputum smear microscopy at partner health care facilities are heat-inactivated before being presented to the rats. Samples which were initially found TB-negative at the health clinic, but which the rats indicate as TB-positive, are subjected to confirmatory diagnostics using WHO-endorsed methods. Applying this strategy has enabled the rats to increase case detection by around 40% over initial partner clinic results.

Given the similar diagnostic challenges facing brucellosis and TB, and the documented success of sniffer rats for TB-detection, we explored the feasibility of training African giant pouched rats to detect brucellosis. As an initial proof-of-concept, the present study explored if *Brucella* emits a unique odor profile by training sniffer rats to detect heat-inactivated cultures of *Brucella abortus* while rejecting *Brucella-*negative specimens, including heat-inactivated cultures of unrelated bacteria (Experiments 1 and 2). Following this training, rats were tested with more clinically relevant samples that had been spiked with the cultured bacteria (Experiment 3). We then assessed rat scent detection accuracy by comparing their sensitivity and specificity to existing diagnostics. Finally, we discuss how developing scent-based detection of brucellosis could provide a novel and cost-efficient diagnostic tool to augment or replace existing methods of detection and ultimately reduce transmission.

## Results

### Exp 1: does ***Brucella*** emit a detectable odor signature?

**Fig. 1:**
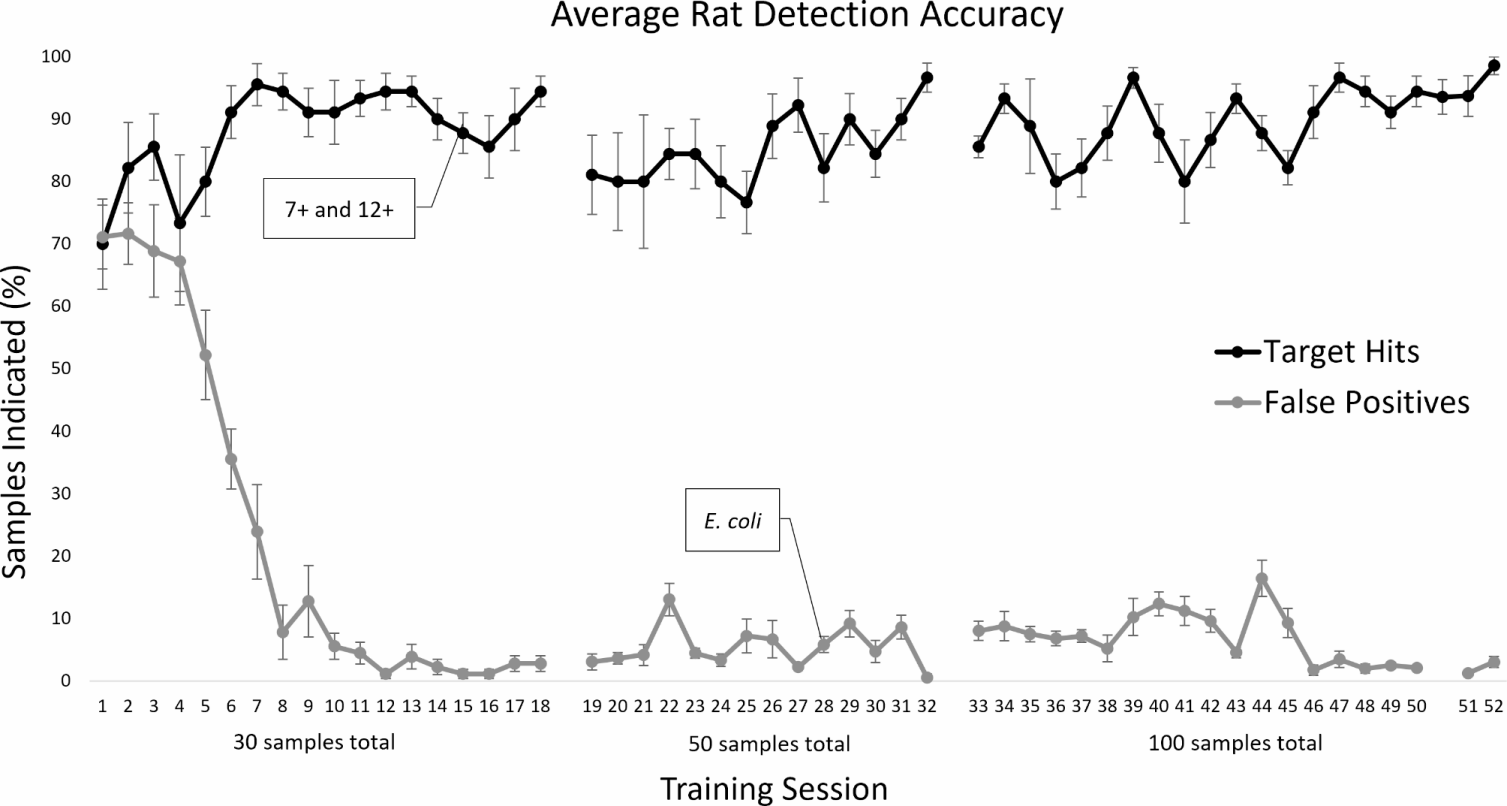
Brucella detection accuracy across training phases. Average percentage of *Brucella-*positive target samples correctly indicated (hit) and false positives (incorrect indications on *Brucella-*negative samples) across all training phases beginning with Phase II (post-indication training). Error bars represent the standard error of the mean (SEM). Rats advanced to Phase IIIa (10-hole discrimination with 30 total samples) on Session 10. New *Brucella*-positive samples (7 + and 12+) were introduced on Session 15. New non-target (*E. coli*) samples were added during Session 28. Sessions 51 and 52 depict the two Baseline refresh training sessions of Experiment 2

All five phases of training were completed within three months. Figure 1 illustrates the average percent of *Brucella-*positive samples correctly indicated (out of 10 samples per session) and the percent of false positives committed on negative samples across discrimination training phases (Phases II through V). After just eight sessions of discrimination training, rats clearly discriminated between *Brucella*-positive and negative samples by indicating significantly more positive than negative samples (*t*(8) = 19.564, *p* < .001). The rats continued to demonstrate significant discrimination when new samples were added across training phases, including different aged cultures (M = 87.78% targets hit, SD = 9.72 versus M = 1.11% of non-targets falsely indicated, SD = 2.20%, *t*(8) = 28.844, *p* < .001) and cultured *E.coli* (M = 82.22% targets hit, SD = 16.41 and M = 5.83% non-targets falsely indicated, SD = 3.75, *t*(8) = 13.98, *p* < .001).

Across the final five training sessions, the average rat correctly identified 93.56% (SD = 0.65) of the *Brucella-*positive samples, including 92.22% of blind targets (SD = 0.109), while correctly rejecting 97.65% (SD = 0.016) of the non-target samples. Detection accuracy for both targets and non-targets was significantly greater than chance (*t*(8) = 19.978, *p* < .001, *t*(8) = -87.932, *p* < .001 for targets and non-targets, respectively compared to a 50% chance hit rate). Importantly, target detection accuracy did not differ significantly across sample types (M = 95.60%, SD = 0.467, M = 92.78%, SD = 0.0795, and M = 92.64%, SD = 0.084 for 7-, 10-, and 12-day old *Brucella* cultures, respectively; *F*(2, 16) = 1.551, *p* = .242). Likewise, no significant differences in false positives were found between non-target samples (M = 2.13%, SD = 0.017, M = 1.78%, SD = 0.018, M = 3.16%, SD = 0.027, and M = 2.33%, SD = 0.011 for 7-, 10-, 12-day *Brucella*-negative cultures, and cultured *E. coli*, respectively, *F*(3, 24) = 2.073, *p* = .130).

To determine if detection accuracy was influenced by prior rat activity, we compared the sensitivity of the first two rats to the last two rats within each session for all 5 sessions. Because female rats always evaluated the samples after all male rats, we included Sex as an additional fixed factor, revealing no significant main effects of either Evaluation Order or Sex, nor interaction between these factors (*p*s ≥ 0.112).

An initial test following training examined if external cues introduced by sample handling and preparation procedures could have guided or otherwise influenced rat detection accuracy by having all samples prepared by a different researcher who was not privy to the routine methods. One rat failed to complete this test and was therefore excluded from analysis. While average sensitivity (M = 92.50%, SD = 0.071) did not change from baseline (*t*(7) = 0.062, *p* = .952) with the average rat continuing to miss fewer than 1 out of 10 *Brucella*-positive samples, there was a slight, though significant, decrease in specificity (M = 95.83%, SD = 0.025; *t*(7) = -2.938, *p* = .022) caused by an increase in false positives to nearly 4 out of the 90 non-target samples presented at test.

### Exp 2: can rats generalize to younger ***Brucella*** cultures?

Experiment 1 established that African giant pouched rats can identify the scent-signature of cultured *Brucella*. While this demonstration included target samples from a range of peak growth culture ages, the amount of bacterium present in clinical samples is highly variable, requiring diagnostic tools to remain accurate across a broader range of bacterial concentrations. Because changes in odor intensity may be perceived as altogether different scents (cf [24].), and to the extent that odor intensity reflects levels of bacteria present, Experiment 2 examined the rats’ ability to identify low concentrations of *Brucella* by introducing cultures that had only been grown for five days.

Although the test conducted at the conclusion of Exp 1 generated a slight increase in false positives, detection accuracy returned to baseline levels during the two sessions of Baseline Refresh Training that immediately followed (M = 2.43%, SD = 0.0139; *t*(7) = -1.010, *p* = .346). Rat sensitivity to accurately indicate *Brucella*-positive samples remained unchanged from the pre-test baseline period (M = 93.13%, SD = 0.1193 post-test baseline; *t*(7) = -0.171, *p* = .869). Excluding the results from one rat that failed to finish the second session further boosted average sensitivity to 97.14%, (SD = 0.039) while leaving specificity unchanged (M = 97.85%, SD = 0.0122). In other words, of the seven rats completing the two sessions with novel samples, nearly every rat correctly identified all 20 *Brucella*-positive samples presented across the two sessions preceding these tests, while committing fewer than 4 false positives among the 180 negative samples.

**Fig. 2:**
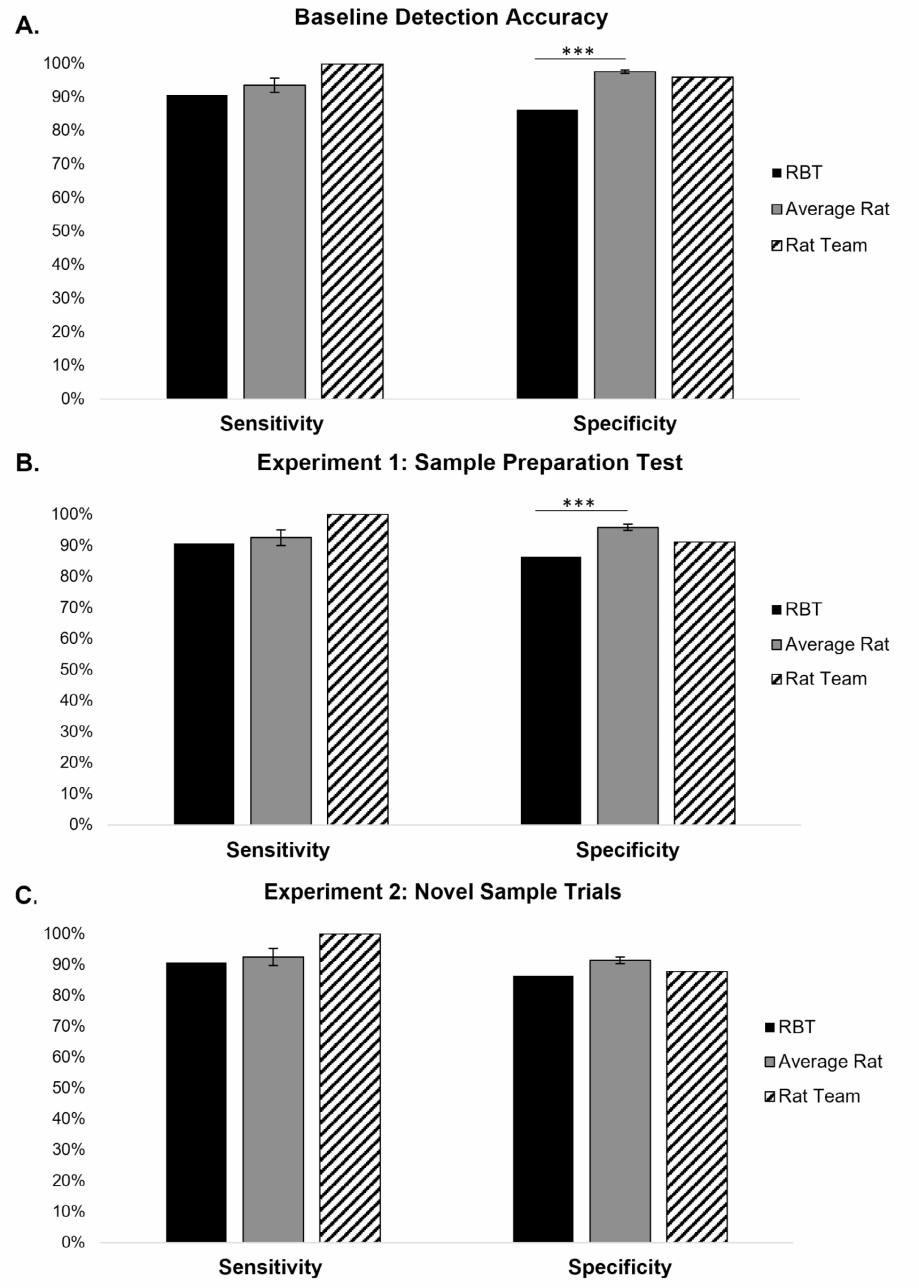
Sensitivity and Specificity of the average rat (grey bars) relative to the reported performance of the Rose Bengal Test (RBT; black bars), and the team of trained rats (using a two-rat cut off; stipled bars) during (**A**) the final 5 training sessions of Experiment 1 (Baseline), (**B**) Experiment 1 Test, and (**C**) Experiment 2 Novel Sample Trials. Error bars for average rat performance represent the standard error of the mean. “***” indicate significant differences (*p* < .001) between groups identified below the horizontal bar

Rat detection accuracy was then assessed for generalization across variants of the trained targets and non-targets by introducing novel samples of both. The same rat that failed to complete the preceding baseline session also failed to evaluate all 100 samples planned during the first test session and was therefore excluded from analysis of this session. Figure 2 displays the average sensitivity and specificity during baseline and novel sample trials. Overall sensitivity to detect *Brucella-*positive samples (including novel samples) was 97.14% (SD = 4.879), which did not differ significantly from baseline (*t*(6) = -0.965, *p* = .372). Importantly, the rats correctly identified all three of the novel 5-day positive samples, including one which was blinded. However, the false positive rate increased from baseline to nearly 6 out of 90 non-target samples (93.49% specificity, SD = 0.0359; *t*(6) = -3.762, *p* = .009) with the majority (94.71%) of these occurring to the 18 blood agar samples (M = 5.53 false positives, SD = 3.04 or 30.71% of these samples). Excluding blood agar samples, the average rat achieved 99.57% specificity (SD = 0.731), representing a slight but significant increase in correctly rejecting the same non-target sample types experienced during baseline (*t*(6) = -3.657, *p* = .011).

During the second test session, sensitivity remained relatively unchanged, averaging 92.50% per rat (SD = 0.116; *t*(6) = 1.333, *p* = .231), but specificity significantly reduced to 89.17% (SD = 0.0104; *t*(6) = 1.333, *p* = .231), driven by a further increase in false positives committed to the 18 blood agar samples (M = 9.52, SD = 2.78), accounting for 87.95% of all false positives. Excluding these samples resulted in specificity that was identical to Session 1 (M = 99.57%, SD = 0.732, *t*(6) = 0.0, *p* > .99).

As at the conclusion of training of Exp 1, we then calculated a univariate analysis of variance (ANOVA) on sensitivity between the first and last rats within each session and by sex, revealing no significant effect of either factor nor interaction (*p*s ≥ 0.084).

### Exp 3: can rats detect cultured ***Brucella*** within field-relevant media?

Although rats successfully identified novel target samples presumed to contain less bacteria and thereby emit fewer related volatiles, they simultaneously committed more false positives to novel non-targets. While generalization from Exp 1 training with *Brucella*-positive samples in the same blood agar media used as novel non-targets during Exp 2 might explain these false positives, it is nonetheless difficult to conclude that successful target detection was driven by generalization of the trained contingencies and scent profiles and not a bias toward indicating any novel sample. We therefore conducted Experiment 3 to introduce truly novel target and non-target samples while simultaneously evaluating external validity and feasibility by using more clinically relevant sample media. Although diagnostic tools exist for the detection of *Brucella* within milk, these methods cannot be applied to non-lactating individuals. Improved detection (including among wild populations) could be achieved if methods existed for reliably testing non-invasive sample media readily available across members of a population. Thus, in Experiment 3 we spiked fecal samples with either *Brucella*-positive or negative culture before presenting them to the rats.

**Fig. 3:**
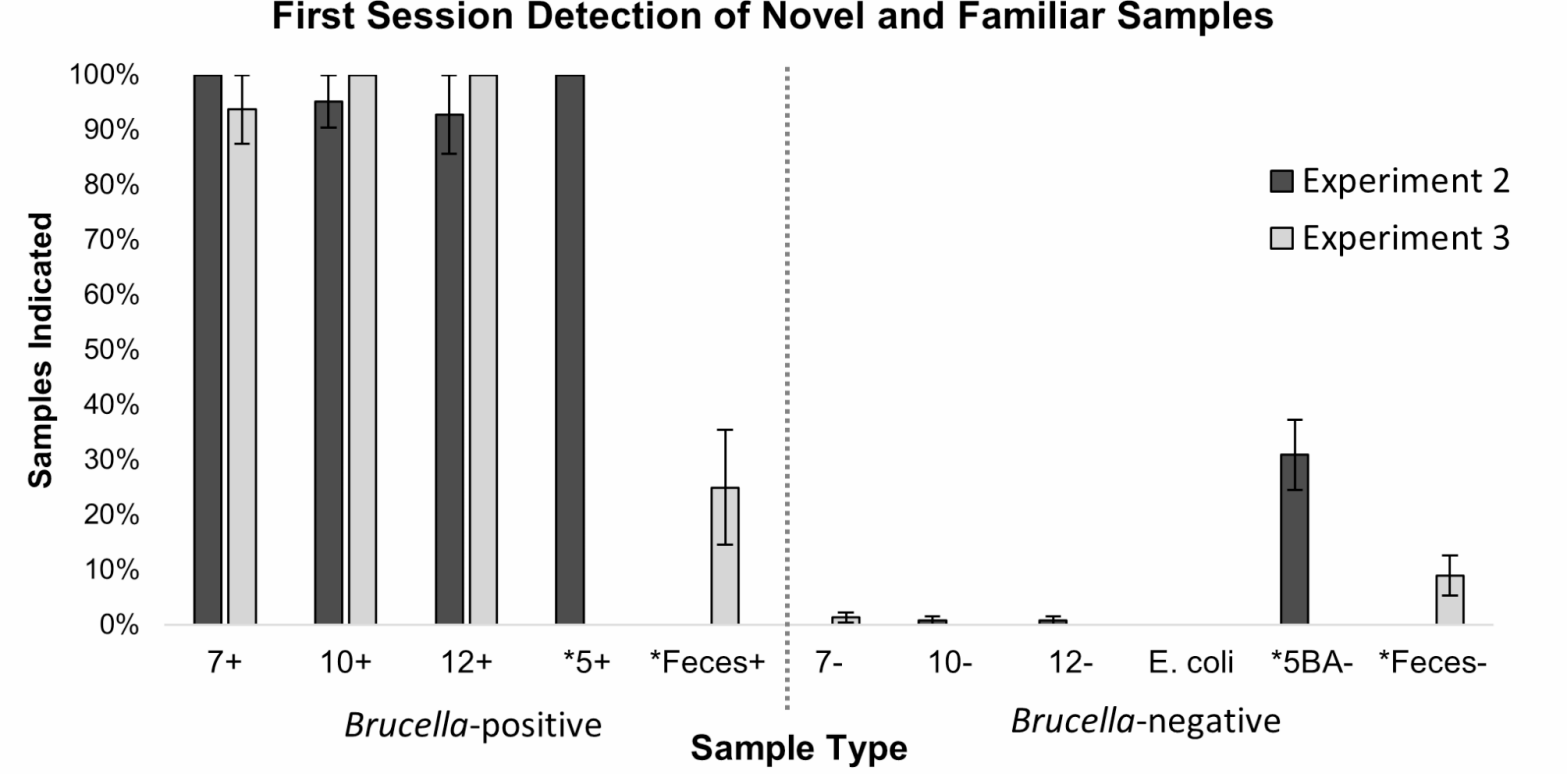
Average percent of samples indicated during the first sessions with novel samples in Experiment 2 and Experiment 3. Error bars represent the standard error of the mean (SEM). Novel samples are denoted with an *. *Brucella*-positive sample types appear to the left of the vertical dashed bar while *Brucella*-negative samples appear to the right. Experiment 2 included novel samples of younger samples (5-day incubation) which were replaced by spiked fecal samples in Experiment 3. Specifically, Experiment 2 session included 10 total *Brucella*-positive samples: 3 samples each of *5 + and 10+ (with one blind sample for each type), and 2 samples each of 7 + and 12+. Additionally, 18 samples of each *Brucella*-negative sample type (7-, 10-, 12-, *E. coli*, and novel *5BA-) were presented. Experiment 3 also included 10 total *Brucella-*positive samples: 3 samples each of 10 + and *Feces+ (with one blind sample per type) and 2 samples each of 7 + and 12 + and 18 samples of each *Brucella-*negative sample type (7-, 10-, 12-, *E. coli*, and *Feces-)

Figure 3 shows the average percent of each sample type indicated by the rats during the first session. Within this session, five of the nine rats successfully found at least one of the three fecal samples spiked with *Brucella*, with some rats finding two, including the blinded trials (M = 0.78 hits out of 3 samples, SD = 0.834). Overall sensitivity decreased from baseline, averaging 76.67% per rat (SD = 0.100; *t*(8) = 4.604, *p* = .002), however there was no change in sensitivity for familiar (non-fecal) samples (M = 98.41%, SD = 0.047, *t*(8) = -1.780, *p* = .113). Notably, despite introducing novel non-target fecal samples during this experiment, omitting the blood agar non-targets from Exp 2 returned specificity to baseline levels demonstrated in both Experiments 1 and 2 (M = 98.03%, SD = 1.907; *t*(8) = -0.401, *p* = .699). Nonetheless, eight of the nine rats falsely indicated at least one of the novel non-target fecal samples, suggesting the rats were not averse to holding their nose in proximity to these samples.

One rat failed to evaluate all 100 samples planned for each session during three of the four following sessions and was therefore excluded from analysis. A repeated measures ANOVA computed across the five test sessions revealed no significant differences in sensitivity (*F*(4, 28) = 1.423, *p* = .252), but significant variance in specificity across these sessions (*F*(4, 28) = 3.730, *p* = .015). A second repeated measures ANOVA including the five different non-target sample types (7-, 10- 12-, *E. coli*, and Feces-) revealed main effects of both Session (*F*(4, 112) = 4.178, *p* = .009) and Sample Type (*F*(4, 112) = 5.170, *p* = .003), but no interaction between these factors (*p* = .663). *Post-hoc* paired-samples *t*-tests were then computed using a Bonferroni correction for multiple comparisons, thereby adjusting alpha to 0.01. This revealed that overall specificity significantly differed during Session 5 (M = 95.14%, SD = 3.082) compared to Session 1 (M = 98.06%, SD = 2.037; *t*(7) = 3.482, *p* = .010) and Session 2 (M = 97.92%, SD = 3.33; *t*(7) = 5.000, *p* = .002). A slight, though non-significant difference (after adjusting alpha) was also found between Session 5 and Sessions 3 and 4 (*t*(7) = 2.393, *p* = .048 and *t*(7) = 2.457, *p* = .044, respectively). No other differences were found (*p*s > 0.135). When applying the same *post hoc* method for comparing sample types, no differences were found (*p*s > 0.049); however, the main effect was likely driven by the greater rate of false positives committed to Feces- samples (Fig. 3; M = 9.70%, SD = 0.106) than all other non-target sample types (M = 1.02%, SD = 0.007 for 7-, M = 1.90%, SD = 0.0148 for 10-, M = 0.75%, SD = 0.009 for 12-, and M = 1.18%, SD = 0.008 for *E*. coli samples).

Nonetheless, a repeated measures ANOVA computed on the percent of novel fecal samples indicated by the rats as containing *Brucella* across the five test sessions revealed main effects of both Sample Type (Feces + and Feces-, *F*(1, 7) = 16.763, *p* = .005) and Session (*F*(4, 28) = 5.903, *p* = .001), as well as a Sample Type X Session interaction (*F*(4, 28) = 5.108, *p* = .003). Although there was no significant difference in the percent of Feces + and Feces- samples indicated during the first two sessions (M = 25.00%, SD = 0.2955 and M = 20.83%, SD = 0.2480 for Feces + during days 1 and 2, respectively versus M = 9.03%, SD = 0.1026 and M = 9.03%, SD = 0.1026 for Feces- during the same sessions; *t*(7) = 1.707, *p* = .132 and *t*(7) = 1.553, *p* = .164), the rats showed evidence of learning to successfully discriminate between these sample types by the third session by correctly indicating more Feces+ (M = 54.17%, SD = 0.3536) than Feces- samples (M = 13.89%, SD = 0.2262; *t*(7) = 4.072, *p* = .005). While this result does not rule out the possibilities that rat detection behavior was initially influenced by novelty or a reduced odor signal to noise ratio introduced by the greater volatiles presumably present in fecal versus agar media, it does suggest scent detection rats can quickly (in as few as three sessions) overcome these challenges to reliably detect *Brucella* within a more clinically relevant media.

To explore if rat detection performance was influenced by extraneous cues introduced by the behavior of other rats evaluating the same samples earlier in the same day, we computed a univariate ANOVA of sensitivity between the first two rats and last two rats of both sexes to evaluate the samples. Because one female rat was excluded from analysis of Sessions 2–5, the sensitivity of only the first and last female was included during these sessions. While there was no main effect of Evaluation Order (*F*(1, 28) = 1.297, *p* = .264), there was a significant interaction between Sex and Evaluation Order (*F*(1, 28) = 9.623, *p* = .004) which may have been driven in part by the reduced number of females overall and their significantly lower sensitivity (M = 64.17%, SD = 0.151) compared to males (M = 78.00%, SD = 0.128, *F*(1, 28) = 10.622, *p* = .003) during these sessions. Post hoc comparisons applying a Bonferroni correction for multiple comparisons revealed male rats evaluating samples that had already been sniffed by other (male) rats earlier in the day demonstrated significantly *lower* sensitivity (M = 69.00%, SD = 0.088) than the males that had preceded them (M = 87.00%, SD = 0.095; *t*(18) = 4.409, *p* < .001), contrary to what would be predicted if the rats’ detection behaviors were influenced by cues introduced to the target samples through the process of evaluation. Similarly, the first female rats to evaluate samples (who always evaluated the samples after males) had significantly lower sensitivity (M = 60.00%, SD = 0.189) than the first males to evaluate the same samples. Female rats were otherwise unaffected by Evaluation Order and no significant differences in target detection sensitivity were found between the last male and last female rats.

## Discussion

Following just three months of training with cultured samples in Experiment 1, the average individual rat missed only 3 out of 50 *Brucella*-positive samples, including nearly all blinded samples (averaging greater than 9 out of 10 samples). The same rats correctly rejected more than 439 of the 450 negative samples presented across five evaluation sessions. Comparing the rats’ average detection accuracy to the most commonly used brucellosis diagnostic test, the Rose Bengal Test (RBT; Fig. 2) with a 89.6% sensitivity and 84.5% specificity [25], the average rat demonstrated comparable sensitivity (using *t*(8) = 1.172, *p* = .275) and superior specificity (*t*(8) = 178.598, *p* < .001). Trained scent detection rat accuracy could be further bolstered by adopting a rat team detection strategy [21] rather than relying on the performance of each individual rat. With this strategy, a defined number of rats are required to indicate the same sample for it to be considered correctly hit (*Brucella*-positive) or falsely indicated (negative samples). Applying a two rat cut-off (in which two or more rats out of the team of seven needed to indicate the same sample) bolstered overall rat detection accuracy to reach 100% sensitivity and 96% specificity (18 false positives).

Although the test conducted at the conclusion of Experiment 1 revealed a slight, though significant increase in false positives, applying the published specificity of RBT [25] as the reference value in a single sample *t*-test revealed that the rats still significantly outperformed this widely accepted diagnostic test (*t*(7) = 104.443, *p* < .001; Fig. 2b). As with the conclusion of training, adopting a rat team strategy improved test sensitivity to 100% of the 10 *Brucella*-positive samples (including two blind samples) while generating only eight false positives out of the 90 *Brucella*-negatives samples. The rat team remained equally accurate (100% sensitivity and 96.1% specificity across 200 total samples) during the baseline of Experiment 2, in which similar samples were presented. In other words, inter-rat agreement between at least two rats correctly identified all 20 *Brucella*-positive samples and correctly rejected 173 of the 180 negative samples. Importantly, the rat team remained 100% sensitive when novel, younger *Brucella*-positive samples were introduced, suggesting they readily generalized across target concentrations. Although false positives among *Brucella*-negative samples increased during this test, all of these were committed to the newly introduced blood agar medium. That is, the rat team achieved 100% specificity among all previously trained non-target samples.

It’s worth noting that while *Brucella*-negative blood agar was novel to the rats during this test, blood agar had previously served as media for *Brucella*-positive samples which the rats were rewarded for indicating early in training. It is therefore possible that the increased false positives generated by these samples was driven by the rats’ prior association between the blood agar and reinforcement. That is, if the rats recognized the blood agar as a scent they had previously encountered, then we might expect them to respond to these samples as *Brucella*-positive given this prior training history. Indeed, others (cf [26].) have demonstrated that African giant pouched rats are not only capable of remembering previously learned scent targets, despite long delays and intervening training with unrelated scent samples, they can also simultaneously search for more than one scent target. Experiment 3 therefore introduced truly novel target and non-target samples with greater clinical relevance to further evaluate rat scent detection accuracy. Applying the same two-rat cut-off team strategy to the first session of Experiment 3 resulted in 90% sensitivity with the only missed sample being the very first encounter of the novel fecal sample spiked with *Brucella*-positive culture solution. Within this same session, the team falsely indicated only three samples, all of which were from the novel category. Moreover, across the entire five sessions, the rat team only missed three of the 50 *Brucella-*positive samples (94.0% sensitivity), all of which were presented in the novel medium (15 in total) and demonstrated 100% sensitivity during three out of the five sessions.

Several diagnostic tests are available for human and non-human brucellosis with different sensitivities and specificities. RBT (the one that was compared with the rats in the current study) is widely used and recommended by WHO and WOAH as a screening test [27]. Our results suggest trained rats can achieve better specificity than RBT contrasts with the findings of a study comparing RBT to five commercial serological tests available in Tanzania [28]. This result suggests scent detection rats could provide better specificity than many serological tests available in Tanzania or other resource-poor areas without a compromise to sensitivity. However, further research would be needed to make the direct comparison between such serological tests using indirect and detect antibody detection to the scent detection of volatile molecules emitted by the bacteria, as in our study.

This study has several limitations, First, although there is growing scientific interest in using scent detection animals for disease detection [29], widespread application of animal-based diagnostics have not been widely accepted, validated, or implemented by the medical community. Further research is needed to empirically establish the diagnostic accuracy of scent detection animals across diseases and clinical environments, as well as their cost-efficiency and implementation feasibility. These factors will be especially relevant to resource-poor areas where few alternatives may exist. Secondly, although our analyses suggested that the order in which rats evaluate samples has little to no consistent effect on their sensitivity to accurately detect *Brucella*-positive samples (as would be expected if the rats had learned to rely on extraneous cues introduced by preceding rats), it remains possible that rats can perceive samples which have been sniffed longer by other rats and use this perceived difference to guide their discrimination behavior. Further research is needed to ensure this possibility is well controlled for in all scent detection scenarios where more than one animal evaluates the same material in sequence. Finally, as a proof-of-principle, the rats were trained with cultured samples. It remains unclear to what extent these samples resemble clinical samples and if the apparent volatile signature of *Brucella* culture could translate to these field-relevant media.

## Conclusion

The availability and use of novel and robust tests for the diagnosis of brucellosis both in non-human and human animals are paramount to minimizing the impact of the disease, including control efforts. The greater the test accuracy, the lower the risk of delays in diagnosing true cases and, consequently, reduced treatment delays and spread of infection [30, 31]. Collectively, the results presented herein indicate scent detection rats can be trained to reliably discriminate *Brucella-*positive and negative cultures across a variety of media. With accuracy that rivals or surpasses RBT, our results suggest trained rats could serve as a valuable, novel method for the detection of *Brucella* infection.

## Methods

### Samples

Cultured *Brucella* were prepared in a laboratory located on the campus of SUA and were brought to the training facility daily. The *Brucella* used in the present study were obtained from a previous project that collected samples from Kitengule Ranch, located in Kagera region, Tanzania. A total of 11 sample types were used during this project; eight were used during initial training while three were reserved for testing experiments that followed (Table 1). Individual samples were prepared the morning of their use. After preparation, the containers were labeled (ID and type, e.g., 10 + or 10-, see Table 1), placed in a plastic bag, and transported to APOPO training facility via a cool box. After each training session, all samples were collected in autoclavable bags and returned to the facility where they were autoclaved for 45 min before disposal.

**Table 1:** Samples used during rat training and tests. All samples were heat-inactivated prior to behavioral sessions. *Brucella*-positive samples served as scent targets for rats, while all *Brucella*-negative samples served as non-reinforced controls

Sample	*Brucella*	Experiment	Description
5+	Positive	Exp 2: Novel trials only	5-day old *Brucella* culture isolates with nutrient agar
7+	Positive	Exp 1, 2, and 3	7-day old *Brucella* culture isolates with nutrient agar
10+	Positive	Exp 1, 2, and 3	10-day old *Brucella* culture isolates with nutrient agar
10BA+	Positive	Exp 1: Phase I only	10-day old *Brucella* culture isolates with blood agar media
12+	Positive	Exp 1, 2, and 3	12-day old *Brucella* culture isolates with nutrient agar
7-	Negative	Exp 1, 2, and 3	Blank nutrient agar incubated for 7 days
10-	Negative	Exp 1, 2, and 3	Blank nutrient agar incubated for 10 days
12-	Negative	Exp 1, 2, and 3	Blank nutrient agar heat inactivated after 12-day period
*E. Coli*	Negative	Exp 1, 2, and 3	*E. coli* culture in nutrient agar
5BA-	Negative	Exp 2: Novel trials only	5-day old blood agar media
Feces+	Positive	Exp 3	fecal sample spiked with 10-day old *Brucella* -positive solution
Feces-	Negative	Exp 3	fecal sample spiked with 10-day old *Brucella* -negative solution

For the preparation of *Brucella* positive samples, isolated *Brucella* bacteria from placental and milk samples was cultured in Farrell’s medium, as described by Mathew et al. [30] The plates were observed for any growth before and after harvesting for training samples beginning on Day 3 through Day 14; those that did not show any growth after 12 days were discarded. After the pre-determined incubation period, isolates from the growth were picked using sterile wire loop. Ten colonies were mixed with 10 mls of distilled water and the resulting solution was heat inactivated at 56 °C for 3 h in a water bath and then allowed to cool. To confirm this process effectively inactivated the zoonotic pathogen, aliquots of the solution were inoculated in growth medium for up to ten days and monitored for any growth before being discarded. On the day of sample use for rat evaluation, 500 μl of the inactivated solution was pipetted into a sample container filled with 5 mls of either nutrient or blood agar (see procedures below).

*Brucella* negative samples were handled identically to positive samples, including culture conditions, heat inactivation, and storage, but contained no *Brucella* bacteria. For their preparation, 10 mls of distilled water was mixed with 10 loopfuls of Farrell’s medium, incubated both aerobically and anaerobically and 500 μl of the solution was pipetted into a sample container filled with 5 mls of nutrient or blood agar on the day of their use.

Specimens from ten pre-grown colonies of *Escherichia coli* (*E. coli*) were mixed with 10 mls of distilled water to form a solution. The resulting solution was heat inactivated at 56 °C for 3 h in a water bath and then allowed to cool. On the day of their use, 500 μl of the *E. coli* solution was pipetted into sample containers filled with 5 mls of nutrient agar.

Spiked fecal samples were introduced in Experiment 3. Fecal samples were collected in stool containers from animals that screened *Brucella*-negative using RBT. Samples were collected from both Kitengule Ranch and from Magadu Farm at SUA and were kept refrigerated at 4 °C. A spatula was used to pick feces which was mixed with 1 ml of distilled water and the resulting solution was placed into a sample container. These samples were then spiked with 500 μl of either *Brucella*-positive or *Brucella*-negative solution (incubated for 10 days, as described above) and pipetted into each container before they were transported for rat evaluation sessions later the same day.

### Subjects

Nine (5 male) African Giant Pouched rats (*C. ansorgei*) averaging 3.7 years at the start of the experiment were sourced from APOPO’s rat colony in Morogoro, Tanzania. Sample size was determined based on prior work [23]. One (female) rat failed to complete the test at the conclusion of Experiment 1 and was therefore excluded from Experiment 2. All rats had similar experiences serving as subjects in prior experiments with unrelated odors. Rats were single- or pair-housed in cages that contained clay pots for sleeping, wood shaving substrate, and a chewing/climbing stick made from untreated wood. Home cages were located within a vivarium maintained between 21 and 34° C with a natural light:dark cycle of approximately 12:12 h. All sessions were conducted during the light period. Rats were maintained at their *ad libitum* weight by providing opportunity to earn up to 36 banana-flavored pellets (5 g, 5TCY OmniTreat^™^) during experimental sessions, which was supplemented at the end of the working day with 20 g of commercially available rodent chow (Specialty Feeds, Glen Forrest, Western Australia). Prior to weekends, rats were fed 65 g of chow along with locally sourced fresh foods including 10 g of sundried sardines and one produce item, (e.g., mango, avocado, banana, etc.). Rats always had access to clean drinking water while in their home and transport cages.

Prior to the start of the experiment, all rats were pre-screened for *Brucella* infection. Blood samples were collected from the tail vein of each rat using plain vacutainer tubes, and then centrifuged to obtain a serum. The resulting serum samples were subjected to a Rose Bengal Test (RBT) for *Brucella* antibodies according to the procedure recommended by the WOAH [4]. This test was repeated after the project was completed. Both pre- and post-screening tests generated negative results.

At the conclusion of all experiments, rats were retired to APOPO’s colony in Morogoro, Tanzania where they could serve as subjects in unrelated experiments or join APOPO’s breeding program. While in retirement, rats continued to be housed, as described above, but with *ad lib* access to the same foods. Rats continued to receive daily health and welfare house checks by APOPO staff and weekly veterinary inspections. Additionally, rats were given at least 15 min to explore a shaded, ventilated outdoor enclosure containing a variety of enrichment items (custom-made running wheel, bamboo poles and ramps, sisal rope, etc.) three or more times weekly.

### Apparatus

**Fig. 4:**
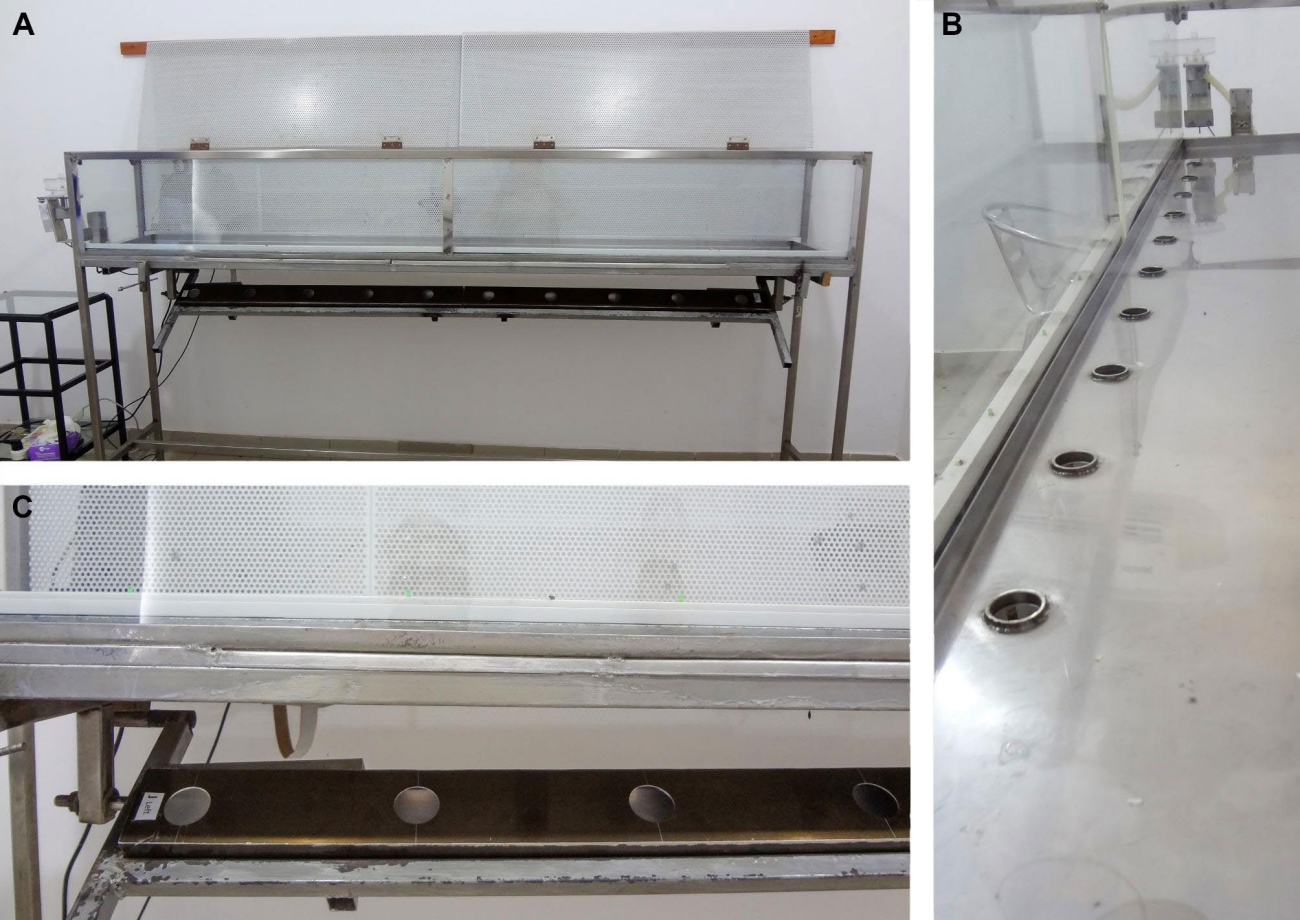
Photographs of the front (**A**) and side (**B**) view of the rectilinear scent evaluation apparatus. Cups containing culture sample solutions were placed within bars (**C**) that could be swung up and locked to position the sample beneath the holes along the floor of the apparatus. Rats were trained to walk from one side (beginning near the food pellet dispenser shown to the left in A) to the other, sniffing each sample in sequence as solenoids below the floor slid a metal plate to uncover each sample. Light emitting diodes (LEDS; pictured to the left of the floor holes along the white rail in B) illuminated when a hole was uncovered. Infrared emitting photobeams and sensors positioned within each hole registered when and for how long the rat inserted its nose. Flavored food pellets were automatically dispensed when the rat held its nose within a hole containing a *Brucella*-positive sample for the prescribed duration

A custom engineered line cage (similar to apparatus described and illustrated elsewhere [26, 32] and as shown in Fig. 4) measuring 247 × 41 × 34 cm mounted on four 96 cm high legs, with hinged top panels and glass walls was used for all experiments. Ten circular holes, measuring 30-mm in diameter, were evenly spaced on the floor of the cage. Each hole was fitted with a through-beam (infrared) photoelectric sensor (ENV-254, MedAssociates) and covered by an aluminum plate that slid open via underlying solenoids. Breaks in the infrared beam (when the rat inserted its nose into the hole) were accompanied by a continuous beep as auditory feedback to the rat. The first hole opened at the start of the session and each subsequent hole opened after the infrared beam was broken in the hole immediately preceding it. Light emitting diodes (LEDs) located inside the Perspex wall of the cage alongside each hole turned on and off in correspondence with the opening and closing of the holes. The aluminum plate covering each hole closed 500 milliseconds (ms) after the rat removed its nose from the hole. On the left side of the cage, a pellet dispenser (ENV-203-94, MedAssociates, Georgia, VT) delivered pellets via a 20 cm long plastic tube attached to a 6 × 6 cm square receptacle located flush with the chamber floor. Timing and duration of beam breaks, and delivery of all auditory feedback, and food pellet reinforcement was controlled using custom designed software (MS Visual Basic).

Aluminum cassettes measuring 192 × 8 × 45 cm, with 10, 40-mm diameter holes positioned in correspondence with the 10 holes in the floor of the cage, were loaded with sample containers before the first daily session. Sample containers were 40 ml plastic pots measuring 5.6 cm tall by 3.5 cm wide. The pre-loaded cassettes fit into a hinged bracket that swung up and locked into position underneath the cage.

The interior walls, floor, and scent holes of the apparatus were wiped with a clean paper towel and 70% methylated spirits following each rat’s evaluation session. If a rat urinated or defecated during the session, all cage holes were immediately closed to prevent sample contamination, the rat was removed from the cage, and the affected area was wiped clean using a paper towel and 70% methylated spirits. The rat was then returned to the cage to resume the session.

### Data analysis

All data were analyzed using SPSS 20 (IBM). Individual rats occasionally failed to evaluate all samples assigned to a daily training or test session. Results from these rats were excluded from analysis, as noted below. Baseline detection accuracy for evaluating test performance was calculated using the last five training sessions in which the specified criterion was met.

### Behavioral procedures

**Fig. 5:**
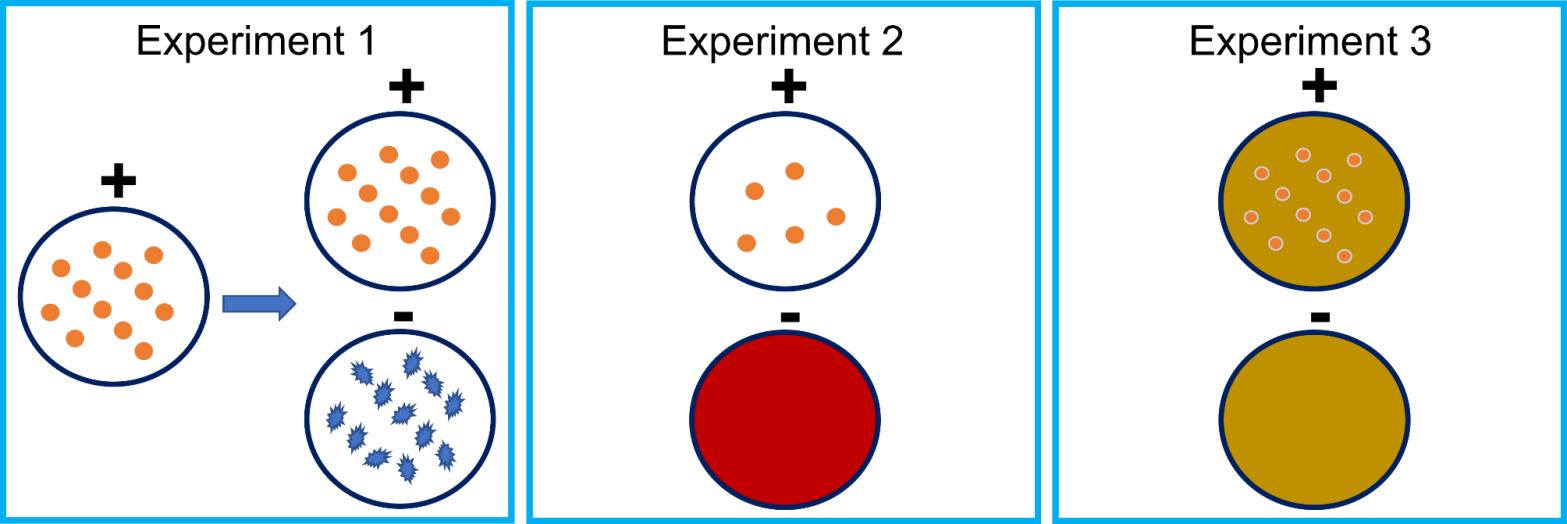
Diagram illustrating the sequence of experiments in which all rats participated. The + indicates target samples for which rats were reinforced for indicating while the – denotes non-target samples. Experiment 1 trained rats to identify the scent of *Brucella* culture in agar then to discriminate this from irrelevant *E. Coli* culture in the same media. In Experiment 2, younger *Brucella* culture was presented to the rats to determine to what extent the odor profile it emitted remained recognizable. To control for any influence of novelty on detection behavior, non-target blood agar media was also introduced. Finally, in Experiment 3, *Brucella-*positive and negative culture solutions were spiked into stool samples collected from animals that had screened negative for brucellosis

All training and test sessions (collectively referred to as evaluation sessions) across experiments were conducted five days per week, excluding weekends and public holidays. We adopted a within-subject design in which each rat underwent identical training (as described below) to determine if rats can be trained to reliably detect the scent of cultured *Brucella* by comparing individual rat behavior in response to target (*Brucella*-positive) and non-target (*Brucella*-negative) control samples (Fig. 5). All rat evaluation sessions lasted a maximum of 30 min with rats completing sessions individually in succession If a rat took more than 30 min to complete a session, it was removed from the cage, the trial at which the rat stopped was noted, and the session was not repeated. If at any time during an evaluation session, three minutes elapsed without a trial being completed due to inactivity or hyperactivity, the rat was removed from the apparatus, returned to its transport cage, and given another opportunity to resume training after the last rat had finished. Repeated sessions started with the trial the rat had previously failed to complete. As a result of this procedure, the sequence in which each rat evaluated the samples could fluctuate from day to day (for example, across 12 test sessions, the sequence of rats evaluating samples changed 19 times); however, male rats always completed evaluation sessions prior to female rats.

Trials within a session were initiated and terminated by the rat’s behavior. When the rat approached the first hole, the trainer used the software to start the session, thereby triggering the first hole to open. When the rat inserted its nose into the hole (termed nose poke), the duration of the beam break (nose poke) was recorded, and the next hole opened. If the duration of the nose poke met or exceeded a pre-determined indication threshold on a hole containing a *Brucella*-positive sample, the response was classified as a “hit” and resulted in reinforcement (delivery of flavored food pellets). There were no programmed consequences if the indication threshold was exceeded over a *Brucella*-negative sample, but the nose poke indication was categorized as a “false positive.” If the indication threshold was not met, the response was categorized as a “miss” when it occurred in a hole containing a *Brucella*-positive samples, or a “correct reject” if the hole contained any *Brucella*-negative sample, including *E. coli* controls. The indication threshold for all rats was initially set at 2000 ms but was adjusted for each rat depending on individual performance according to the guidelines described below. When all samples in the cassette had been evaluated, trainers removed the cassette and inserted the next one, while the rat remained in the cage.

#### Exp 1: does ***Brucella*** emit a detectable odor signature?

Training occurred in five phases with progression through the phases determined by the performance of the entire team of rats. Criteria for phase advancement was pre-determined using the published sensitivity and specificity of current diagnostic methods as a guide [31]. Phase advancement never occurred after a weekend or holiday to ensure performance was not impacted by the break in training.

##### Phase I: indication 12 samples (8 sessions)

A clear Perspex glass divider was positioned between holes three and four, thus restricting access to only the first three holes. Rats were presented with 12 *Brucella*-positive samples (6 each of 10 + and 10BA+) per session and were required to meet the indication threshold to receive reinforcement. To advance to Discrimination Training, 7 of the 9 rats were required to correctly indicate (exceed threshold) on at least 10 samples within a single session.

##### Phase II: 3-Hole discrimination 30 samples (12 sessions)

The divider remained in the same position, however non-target samples were introduced. Rats were presented with 30 samples per session, including 10 samples of 10 + and 20 *Brucella*-negative samples (10-). At the start of this phase, the indication threshold was set to 1500 ms for all rats, which served as the minimum threshold for the rest of training. If, after four days of training, a rat committed more than 10 false positives for two consecutive sessions, the indication threshold was increased by 500 ms. Indication thresholds continued to be adjusted following these procedures for all subsequent phases. At least 7 rats were required to hit at least 9 of the 10 + target samples (≥ 90% sensitivity) and commit no more than 5 false positives (≥ 75% specificity) for two consecutive training sessions to advance to the next phase.

##### Phase IIIa: discrimination 30 samples (6 sessions)

The Perspex divider was removed, allowing access to all 10 sample holes within the cage. As with Phase II, rats were presented with 30 samples per session, including 10 samples of 10 + and 20 *Brucella*-negative samples (10-). After two consecutive sessions in which at least 7 rats hit at least 9 targets while committing no more than 5 false positives, target and non-target samples of the 7- and 12-day samples were introduced.

Rats continued to be presented with 30 samples per session; however, the 10 positive samples included four samples of 10 + plus 3 samples each of the new 7 + and 12 + targets. Likewise, the 20 negative samples included 8 samples of 10- and 6 samples each of the new 7- and 12- non-targets. At least 7 rats were required to hit 9 positives and commit no more than 5 false positives for two consecutive sessions before advancing to Phase IIIb.

##### Phase IIIb: discrimination 50 samples (9 sessions)

Rats were presented with 50 samples per session, including 10 positive samples (4 samples of 10 + and 3 samples each of 7 + and 12+). Two blind trials were included per session. These samples were coded as negative so even the trainers conducting the sessions were unaware that these samples contained *Brucella*. Blind trials served two purposes: (1) they ensured that the rats’ detection behavior was driven by the odors emitted by the samples themselves, rather than any extraneous cues, and (2) they prepared the rat for potential future screening work in which the true status of any given sample is unknown until after the rat has performed the evaluation. In this scenario, the rat cannot be rewarded for indicating the sample. Thus, blind trials accustom the rat to working under conditions of partial reinforcement in which some correct indications are not reinforced. Two different blind samples were chosen at random each day. Additionally, 40 negative samples were included in each session, including 14 samples of 10- and 13 samples each of 7- and 12-.

When at least 7 rats hit at least 9 targets (≥ 90% sensitivity) and had no more than 10 false positives (≥ 75% specificity) for two consecutive days, a novel negative sample (*E. coli*) was introduced.

##### Phase IV: *E. coli* controls (5 sessions)

The inclusion of cultured *E. coli* samples ensured that the rats learned to specifically identify *Brucella* bacteria rather than any bacteria in comparison to bacteria-free culture medium. *E. coli* controls therefore served a crucial function in determining to what extent *Brucella abortus* emits a unique odor signature. Rats continued training with 50 samples per session, including 10 *Brucella*-positive targets (4 samples of 10 + and 3 samples each 7 + and 12+) from which two different blind samples were randomly chosen daily. The 40 negative samples included 9 samples of 10-, 8 samples each of 7- and 12-, and 15 *E. coli* samples. To advance to the next phase, at least 7 rats were required to hit at least 9 positives while committing no more than 10 false positives for two consecutive days.

##### Phase V: discrimination 100 samples (19 sessions)

Rats were presented with 100 total samples per session: 10 positive (4 samples of 10 + and 3 samples each of 7 + and 12+; two different blinds chosen randomly each day) and 90 negative samples (two types from among 10-, 7-, 12-, and *E. coli* randomly selected each day to have 22 samples while the two remaining types each had 23 samples). After at least 7 rats correctly indicated at least 9 positive samples and committed no more than 12 false positives for at least 4 out of 6 consecutive sessions, the rats advanced to test.

##### Test (1 session)

This test ensured extraneous cues inadvertently introduced during sample preparation were not guiding rat detection by enlisting a naïve researcher to prepare all samples. The new sample preparer was only instructed how to prepare the samples but not in which order. This effectively randomized potential cues that might have resulted from the training sample preparer or method of preparation during training.

#### Exp 2: can rats generalize to younger cultures?

Experiment 2 began the day immediately following Experiment 1 Test.

##### Baseline (2 sessions)

To ensure the Experiment 1 Test did not disrupt baseline scent detection performance, two sessions identical to Experiment 1, Phase V Discrimination Training were conducted. At least 7 rats correctly indicated at least 9 positive samples and committed no more than 12 false positives during these two sessions.

##### Novel samples (2 sessions)

A novel *Brucella*-positive sample was introduced to the rats while blood agar media (heat inactivated) was also re-introduced, this time as an un-spiked *Brucella*-negative sample. The novel *Brucella-*positive samples were prepared as before, except they contained *Brucella* culture harvested after just 5 days of incubation (5+). As with Experiment 1, a total of 10 targets were presented, including 3 novel target samples (5+, with one blind sample), 3 samples of the previously trained 10+ (with one blind sample) and 2 samples of each remaining familiar target (7 + and 12+). Among the 90 non-target samples, rats encountered 18 samples of each familiar type (7-, 10-, 12-, and *E. coli*) plus 18 samples of un-spiked blood agar (5BA-). Although rats had previously encountered *Brucella*-positive samples with blood agar media during Phase I of Experiment 1, *Brucella*-negative blood agar was novel to the rats and served as control for potential novelty bias that might otherwise confound interpretation of 5 + detection accuracy.

#### Exp 3: can rats detect cultured Brucella within field-relevant media?

Each evaluation session included 100 total samples comprised of 2 samples each of familiar *Brucella*-positive targets (7+, 10+, 12+), plus one blind sample randomly selected from among these types. Additionally, 3 fecal samples (including 1 blind sample) were spiked with *Brucella*-positive culture solution (Feces+) to serve as novel targets. Among the 90 non-target samples, rats encountered 18 samples of each familiar control type (7-, 10-, 12-, and *E. coli*) plus 18 novel fecal samples spiked with the *Brucella*-negative control solution (Feces-). Feces- samples not only controlled for any inherent bias for or against novelty but also any potential bias for the fecal samples themselves. Sessions were conducted for five consecutive days.

## Data Availability

The datasets generated and analyzed during the current study are available from the corresponding author on reasonable request.

## List of abbreviations

BA: Blood Agar
CFT: Complement fixation test
ELISA: Enzyme-linked Immunosorbent Assay
LED: Light Emitting Diode
ms: Milliseconds
PCR: Polymerase Chain Reaction
RBT: Rose Bengal Test
SAT: Serum Agglutination Test
